# A Short Overview of Biological Fuel Cells

**DOI:** 10.3390/membranes12040427

**Published:** 2022-04-15

**Authors:** Ivan Vito Ferrari, Luca Pasquini, Riccardo Narducci, Emanuela Sgreccia, Maria Luisa Di Vona, Philippe Knauth

**Affiliations:** 1 University of Rome Tor Vergata, Department Industrial Engineering, International Laboratory “Ionomer Materials for Energy”, 00133 Rome, Italy; riccardo.narducci@uniroma2.it (R.N.); emanuela.sgreccia@uniroma2.it (E.S.); divona@uniroma2.it (M.L.D.V.); 2 Aix Marseille University, CNRS, MADIREL (UMR 7246), International Laboratory “Ionomer Materials for Energy”, Campus St Jérôme, 13013 Marseille, France; philippe.knauth@univ-amu.fr

**Keywords:** biological fuel cell, enzymatic fuel cell, microbial fuel cell, proton exchange membranes, anion exchange membranes, electrochemical performance

## Abstract

This short review summarizes the improvements on biological fuel cells (BioFCs) with or without ionomer separation membrane. After a general introduction about the main challenges of modern energy management, BioFCs are presented including microbial fuel cells (MFCs) and enzymatic fuel cells (EFCs). The benefits of BioFCs include the capability to derive energy from waste-water and organic matter, the possibility to use bacteria or enzymes to replace expensive catalysts such as platinum, the high selectivity of the electrode reactions that allow working with less complicated systems, without the need for high purification, and the lower environmental impact. In comparison with classical FCs and given their lower electrochemical performances, BioFCs have, up to now, only found niche applications with low power needs, but they could become a green solution in the perspective of sustainable development and the circular economy. Ion exchange membranes for utilization in BioFCs are discussed in the final section of the review: they include perfluorinated proton exchange membranes but also aromatic polymers grafted with proton or anion exchange groups.

## 1. Introduction

The inexorably increasing energy demand is a strong motivation to develop sustainable and clean energy systems with the perspective of a circular economy. The world energy demand should increase by almost 30% in the next two decades, with still a lingering dependency on oil and gas (about 50% of the total energy demand). Consequently, a sustained growth of new forms of energy is necessary with the increasing exploitation of renewable energy (wind, solar, and tides). The peak power capacity of these sources is steadily increasing and the need for energy storage and conversion becomes essential, above all for electrical grid stability (due to intermittent renewable energy sources and accidental threats), but also to reduce energy price fluctuations during peak demand and economic losses due to power outages. 

Electrochemical energy storage and conversion (EESC) devices are very promising for their flexibility of utilization and high efficiency [1,2,3,4]. Even if the cost of EESC implementation is relatively high, it is considered essential for the future intelligent network, which should integrate a significant amount of renewable energy resources and provide electricity to hybrid and electric facilities including vehicles [5,6]. EESC presents new opportunities for the protection of the natural and human environment but also challenges involving the materials and conditions to implement them.

Fuel cells (FCs) using fuel sources such as hydrogen, biomass, and biofuels are among the most promising green energy technologies available [2,3,4,7,8,9,10,11].

Biofuel cells (BioFCs) are an alternative to classical FCs by reducing the dependence on expensive and critical raw materials (such as Pt), by reducing the cost of the overall process when wastewater is used, and to realize ecofriendly systems that can be utilized in a biological environment. BioFCs can convert the chemical energy of organic or inorganic compounds using biocatalysts, alternatively microorganisms or enzymes, instead of the traditional inorganic catalyzers in an attempt to reduce the environmental impact [12,13].

BioFC technologies are developed for many applications [12], including the treatment of wastewater [14], water desalination [15], energy production [13], and remote biosensors [16].

The literature described in this short review focuses on the two main BioFCs categories: microbial fuel cells (MFCs) [17,18,19,20] and enzymatic fuel cells (EFCs) [21]. This classification is not exhaustive even if a lot of BioFC technologies can be included in these two categories.

## 2. Microbial Fuel Cells (MFCs)

Recently, MFCs have attracted great attention for the possibility of using different biodegradable substrates as fuel [21,22,23,24]. They can be efficiently applied for energy production and wastewater treatment [14,25,26,27,28,29,30], biosensors for oxygen [16,31], or pollutant removal [22,32]. There are many different kinds of MFCs: for example, mediated, mediator-free, soil-based, etc. The advantages of MFCs include (i) the direct conversion of the fuel into electric energy with a high conversion efficiency; (ii) the efficient operation at room temperature, different from other bioenergy treatments; (iii) MFCs do not need the waste gas treatment step because the main product of the reaction is carbon dioxide, which does not have reusable energy; (iv) the possibility to operate at the cathode only by ventilation without high-pressure gases; and (v) the possibility to be used in areas lacking power infrastructure (construction of MFCs that consume a fuel available in a specific area). Their design, similar to classical FCs, includes an anaerobic anodic chamber and a cathodic chamber, which is usually separated by a membrane ensuring the ion exchange and avoiding the presence of oxygen at the anode that can drastically affect the performances. The anodic chamber, where the microbial metabolism is used to oxidize the substrates, provides the necessary conditions for the growth and electron transfer of microorganisms. MFCs work thus by exploiting the bacterial respiration as a redox reaction where electrons are extracted from bacterial food sources and feed into an electrical circuit to generate power [17,33,34,35,36]. The power generated by MFCs depends on both biological and electrochemical processes. The main influencing factors include the rate of substrate conversion and the anode and cathode hyperpolarization.

A complete list of Microbial Electrochemical Technologies (MET) has been given by Logan et al. [17].

As an example, the cell potential when using acetate with the following conditions: [CH_3_COO^−^] = [HCO_3_^−^] = 4.5 mM, pH = 7, T = 25 °C, pO_2_ = 0.2 bar is E_cell_ = 1.101 V [37].

Anodic reaction: CH_3_COO^−^ + 4H_2_O → 2HCO_3_^−^ + 9H^+^ + 8e^−^ (1)

Cathodic reaction: O_2_ + 4H^+^ + 4e^−^ → 2H_2_O (2)

Although there is great perspective for MFCs, extensive optimizations are required to exploit the maximum microbial potential [38,39,40].

**Figure 1:**
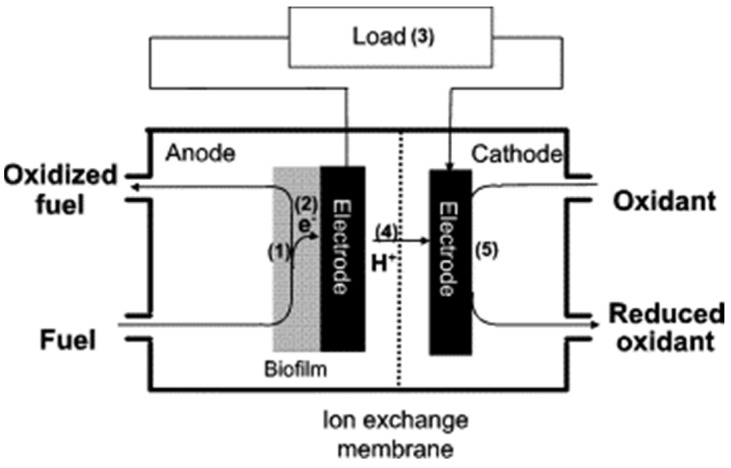
Schematic working of a microbial fuel cell and factors limiting the performance. (1) Anodic catalytic activity, (2) microorganism to electrode electron transfer, (3) load resistance, (4) proton transfer through the membrane to the cathode, and (5) dissolved oxygen concentration and reduction rate at the cathode. Reproduced with permission from Ref. [39].

Kim et al. [41] reported the main limiting factors of MFCs in working conditions, including proton transfer, poor oxygen reduction kinetics, and the influence of various parameters (anodic catalytic activity, fuel diffusion, etc.) on the rate of fuel oxidation (Figure 1).

Pandey et al. [26] studied MFCs operated under a range of conditions including temperature, electron acceptor, electrode surface area, reactor size, and operation time. Gil et al. identified a series of limiting factors for the current generation in MFC including the pH, the load resistance, the electrolyte, and the dissolved oxygen concentration in the cathode compartment [39]. The pH affects the microbial activity; moreover, if the pH is around 9 (in the example of the cited article), it can result in poor proton transfer across the membrane. For the same reason, the choice of the electrolyte is important to avoid any variation of pH in working conditions. A higher load resistance results in a lower Coulombic yield, which is attributed to “*electrons that are consumed in the anode to reduce other electron acceptors*, *such as sulfate and nitrate*, *or oxygen diffused through the membrane*”. The dissolved oxygen concentration determines the maximum amount of electricity generation. Moreover, a high flow of oxygen can result in lower performances, because the microbe immobilization at the cathode can be disturbed. With a low resistance, the proton transfer and the oxygen supply limited the cathode reaction. A mitigation strategy can be the use of a high-strength buffer to reduce the proton limitation [39]. A further limiting factor of MFCs is that they cannot operate at extremely low temperatures because microbial reactions are slow [33]. The output power of an MFC is generally low; it is not sufficient to continuously supply a little device (sensor, transmitter etc.) [31]. A modification of the electrodes, a power management program, or the use of ultracapacitors are the main solutions proposed.

Many studies in the literature are investigating potential solutions to improve the performance of MFCs [34,36,42,43,44,45,46,47]. Kim et al. studied various techniques to enrich electrochemically active bacteria on the electrode: a methanogen inhibitor (2-bromoethanesulfonate) was demonstrated to be able to increase the Coulombic efficiency to 70% [40]. Jang et al. developed a membrane-less MFC, where it was possible to enrich the microbial catalyst by inoculation of an activated sludge [48]. Mitropoulos et al. compared three different generations of MFCs with distinct electron transfer mechanisms. They concluded that the natural mediation properties of sulfate/sulfide species with the reduction in sulfate *Desulfovibrio desulfuricans* is the best solution for the substrate-to-energy conversion efficiency and thus for the power maximization of the device [49].

Recently, the electron storage during the intermittent operation of electroactive biofilms (EAB) has been shown to play an important role in power output and efficiency. Yasri et al. [50] reported the catalytic activity of biofilms grown on the surface of electrodes in aqueous media, which was exploited for the simultaneous remediation of environmental pollutants and energy generation. Ter Heijne et al. [51] investigated the two main mechanisms for electron storage in EAB, namely the storage in the form of polymers and in the form of reduced redox-active compounds. However, it is still unknown how these storage mechanisms contribute to the total storage and what is their dependency on the electrode polarization.

**Figure 2:**
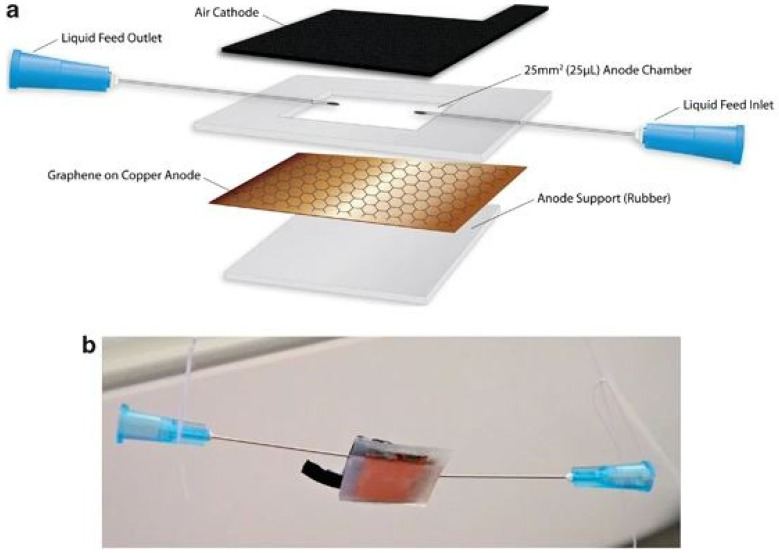
Micro-sized MFC (**a**) schematic and (**b**) digital photograph. Reproduced with permission from Ref. [52].

Looking forward to the miniaturization of these devices, Mink et al. [52] developed a mobile and inexpensive micro-sized device that can be fueled with human saliva (Figure 2). The MFC was fabricated with inexpensive rubber support structures, a multi-layer graphene anode grown on top of copper foil for an efficient current generation, and a sustainable air cathode. The use of a graphene anode generated 40 times more power than using a carbon cloth anode, allowing to produce higher current densities than any previous micro-sized MFC with air cathode.

**Figure 3:**
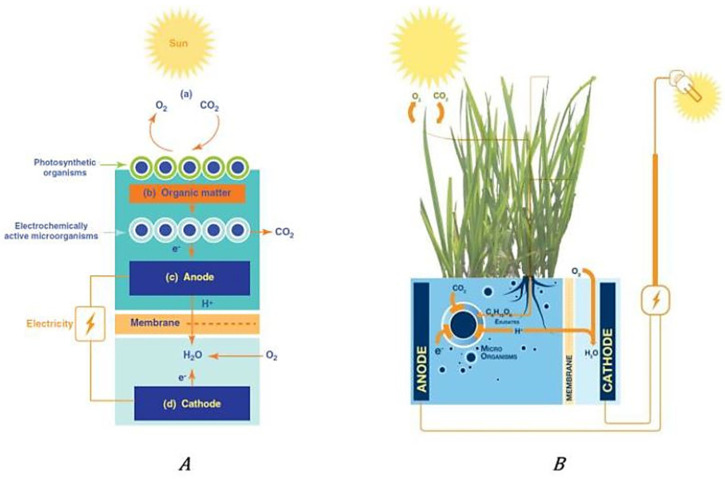
Microbial Solar Cell (MSC) (**A**) of which the Plant Microbial Fuel Cell (P-MFC) **(B**) is a specific type. In the P-MFC, the photosynthetic organisms are plants. Reproduced with permission from Ref. [53].

With the aim to exploit renewable sources of energy, Zaman et al. [53] studied the Microbial Solar Cell (MSC) and the Plant Microbial Fuel Cell (P-MFC), as shown in Figure 3. The P-MFC makes use of natural processes around the roots of plants to directly generate electricity. In this example, three plant species were tested, and especially with *Spartina anglica* and *Arundinella anomala*, it was possible to produce simultaneously bioelectricity and biomass.

The transfer of electrons from microbes to the electrode is a matter of concern for the power optimization. Das et al. [54] and Guo et al. [35] reported a detailed list of microbes used in MFC. Their operation requires exogenous mediators [42] (for example, in the case of *Actinobacillus succinogenes*, *Proteus mirabilis*, *Pseudomonas aeruginosa*, *Streptococcus lactis*, etc.) [55,56,57,58,59] or they can work mediator-less (in the case of *Aeromonas hydrophila*, *Geobacter metallireducens*, *Rhodoferax ferrireducens*, *Klebsiella pneumonia*, etc.) [60,61,62]. Mediator-less MFC have more commercial potential because the mediators are sometimes toxic (such as thionine [43], methyl viologen, humic acid) and are expensive. Metal-reducing and anodophilic microorganisms seems to be the most promising for mediator-less devices [54].

## 3. Enzymatic Fuel Cells (EFCs)

Enzymatic fuel cells (EFCs) are promising sustainable power generation systems for various applications [63,64,65]. Their particularity is the replacement of non-selective metal catalysts, which are currently used in low-temperature FCs, or bacteria with redox enzyme catalysts. Mazurenko et al. [66] discussed the role of redox enzymes and the numerous attempts to convert enzymes into heterogeneous catalysts.

The availability of isolated enzymes has presented the possibility to eliminate the microorganisms from the MFCs and to substitute with individual enzymes with a considerable gain in volumetric catalytic activity but at a substantially higher cost [21]. Isolated enzymes are specific to substrates offering high catalytic turnovers and low overvoltage, but they generally have short lifetimes, even in their natural ambiance. An enzyme is generally capable of catalyzing one specific reaction and theoretically, different enzymes can be associated to create cascade systems. In this last example, continuous or simultaneous reactions are catalyzed by multiple enzymes immobilized on the same electrode: thus, the disposable fuels range is expanded with significant improvements of the output current or voltage and performances. However, structuring this association between enzymes to have specific reactions in the proper order is very challenging [67].

One can resume the advantages offered by the use of enzymes as biocatalysts as follows [65]:

1. Wide possibility of enzymes production based on sustainable biological processes: a huge variety of living organisms can be used as a source to extract enzymes in a renewable way [63].
2. Versatility of catalysts produced for oxidation/reduction in a wide range of substrates, such as sugars, organic acids, alcohols, hydrogen, and others [23,63].
3. Specificity of catalytic redox reactions for their natural substrates that enables in some cases to work in a single chamber cell (i.e., without separation membrane), and make preliminary fuel purification steps unnecessary [65].
4. Possibility to efficiently catalyze reactions under mild and safe conditions at physiological pH, ambient temperature and pressure [63].
5. Reduced cost in comparison with precious metal catalysts [23,68].

**Figure 4:**
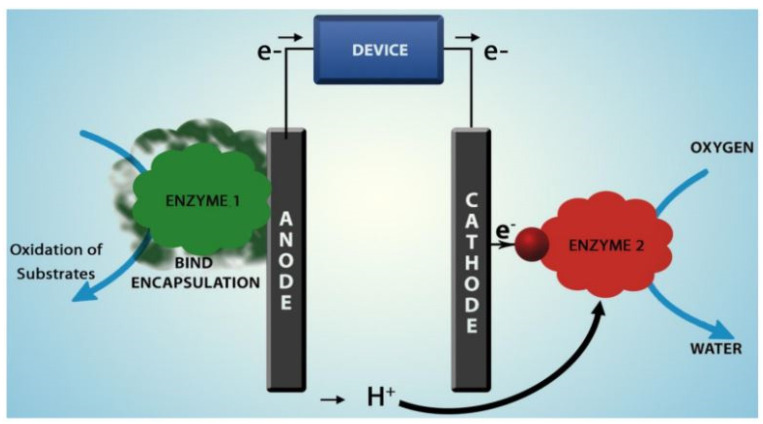
Membrane-less enzymatic fuel cell. Reproduced with permission from Ref. [71].

The design of EFCs can differ from the traditional design of FCs, depending on the application and on the technical choices, but the key constituent elements and the operational mechanism remain the same. Most EFCs are single-chamber designed (Figure 4), but some examples of double-chamber (with an ion-conducting membrane) appeared recently and will be discussed in Section 4. Single-chamber EFCs simplify the design and construction of the cell. Microelectrode materials and electrode preparation technology can be utilized to prepare micro-EFCs with small volume obtaining a potential energy source for electronic devices implanted in the human body. However, as discussed later in the text, some limitations have to be addressed [69].

Aquino et al. [70] summarized the operation of an EFC: appropriate enzymes oxidize the feed fuel on the bioanode and reduce oxidants (usually oxygen or peroxides) to water on the biocathode [63]. An oxidoreductase enzyme can oxidize carbohydrates, alcohols, or even amino acids.

The early EFCs focused on obtaining electrical energy mainly through the oxidation of saccharides, especially glucose and fructose, or other organic fuels such as pyruvate, lactate, methanol and ethanol. The first EFC introduced in 1964 by Yahiro et al. [72] was glucose fueled and utilized glucose oxidase as the anode catalyst [73]. Advantages of this fuel are associated with its wide availability and presence in physiological fluids (for instance in blood or interstitial fluids) and sustainable production by the metabolic processes, which are the most substantial source of energy in many living organisms [67]. Moreover, the interest in glucose as a fuel in EFCs is due to its absence of volatility and toxicity, and its low price combined with a relatively high energy density. More evoluted EFC systems use lactic acid and ethanol as fuel exploiting enzyme electrodes containing auxiliary NADP^+^, such as enzyme electrodes of lactate dehydrogenase [74] and ethanol dehydrogenase [75,76].

Afterwards, the intensive research on the mechanistic understanding of hydrogenases, the key enzyme for H_2_ oxidation in many microorganisms, has made the concept of green energy production through H_2_/O_2_ EFCs possible [70,77]. Hydrogen is significantly smaller than glucose or various other redox mediators, which gives a major advantage in the size of H_2_/O_2_ EFCs [78]. However, in contrast to glucose, which is safe, abundant and easy to handle, the use of hydrogen for EFCs raises problems of H_2_ storage and transport.

More recently, great effort has been put into the improvement of the interfacial electron transfer process between the enzymes and high surface area conductive materials, and to the stability of the enzymes in the typical working conditions, to shift from the proof-of-concept to usable power devices [63,65,66,70,72,77,79,80,81,82,83,84,85,86,87,88,89].

Concerning the electron transfer process and consequently the enzyme immobilization on electrodes, there are still major issues that have to be solved for high energy production [90]. Electrode materials play a crucial role in the electron transfer requiring high conductivity and sufficient porosity to increase the surface where enzymes can be attached. Gold and carbon seem to be the most suitable electrode materials and even simple adsorption on them has often allowed addressing electrochemically many redox enzymes. Nevertheless, an additional functionalization is often needed to manage the enzyme immobilization and exploit the potentiality of the enzymatic catalysts [65,72,80,81,82,83,84,85,86,87,88,89]. Nanostructured carbon-based materials, such as single- or multi-wall carbon nanotubes, carbon nanoparticles, graphene, mesoporous carbon foams, etc., are the most widely used, because they are biocompatible, inexpensive, and capable of boosting the adsorption of enzymes [67]. Recent electrodes, based on hydrogenase enzymes, exhibit promising characteristics such as long-term stability, mW power densities, tolerance to O_2_, and fuel impurities such as sulfides and CO. These properties make hydrogenase electrodes competitive with the Pt catalyst [78,91]. Active research has thus concentrated in recent decades toward the decrease in loading or even the replacement of Pt in FCs [77].

**Figure 5:**
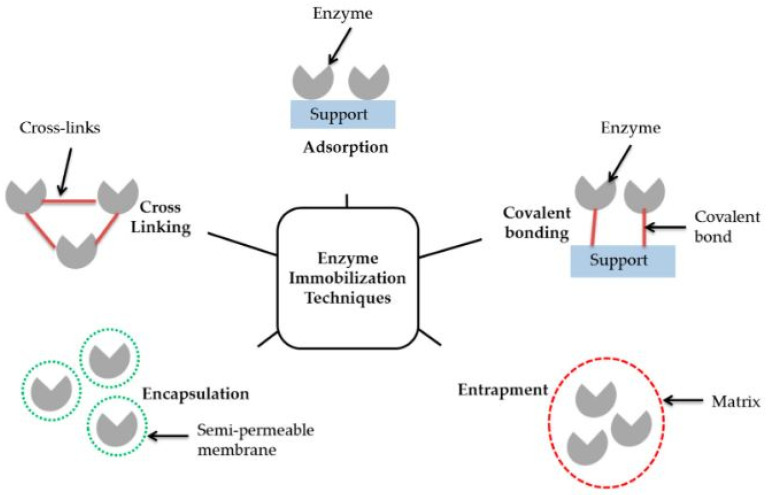
Enzyme immobilization methods. Reproduced with permission from Ref. [94].

A wide range of strategies exists regarding the enzyme immobilization on the electrode surface to provide stable bioelectrodes by avoiding the detachment of biocatalyst and cofactors during operation. The free “in solution” biocatalyst option does not guarantee a proper electron exchange and stability to the system [92]. On the contrary, properly attached enzymes to the electrode by adsorption, covalent bonding, crosslinking, entrapment in polymeric gels, encapsulation (Figure 5), use of nanoparticles, or the combination of these techniques have generally shown improved lifetime of the catalyst and thus of the overall device [93].

An important concern about EFC performance is the polymeric film used for the enzyme immobilization. The polymer matrix should be electronically conductive and should contain functional groups in order to chemically immobilize the enzyme. Many reports show the use of polythiophenes and their derivatives in chemical enzyme immobilization. For example, Korkut et al. have enhanced the power density of the EFC by the improvement of conductivity of poly(3-thiophene acetic acid) (PTAA) by copolymerization with alkyl thiophenes, which is followed by modification with a ferrocene mediator for electron transfer rate improvement [95]. Therefore, polythiophenes can be good candidates as a polymeric matrix in enzyme immobilization because of the presence of carboxylic functional groups and their good conductivity. Recent advances made in the field of bio-nanotechnology (such as different nanostructured materials with a large surface area) allow improving the enzymatic stability and hence the performances [96]. Another technique is the immobilization of the enzymes by micellar polymer encapsulation [92], using modified Nafion ionomer [97,98], Nafion and glutaraldehyde [99], or modified chitosan [100,101].

Concerning the stability of enzymes in working condition, great attention has to be addressed to the EFC operation parameters that should be controlled carefully, since enzymes require strict conditions in terms of temperature and pH, due to their sensitivity and specificity. The working parameters vary from enzyme to enzyme: many enzymes lose their activity at low temperatures, 3–5 °C, or high temperatures, 50–80 °C. For instance, laccase that originates from *Trametes versicolor* and catalyzes the oxygen reduction reaction (ORR) is most active and stable at 30 °C and degrades beyond 50 °C. Glucose oxidase is known to be the most efficient at around 25–30 °C and to deactivate completely at 60 °C. While implantable EFCs operate at temperatures that may be assumed constant, other cells that are used in variable atmospheric applications may suffer from the difficulty of maintaining the operating temperature, leading to the fluctuation in current and power densities [67]. Concerning the acidic or basic environment inside the cell, many studies have reported that physiological pH at around 7.4 is the most appropriate condition for enzymatic performance, since enzymes mainly function in living organisms. However, fluids with natural acidic properties such as fruit juices or gastric fluid can also be utilized in EFCs. EFCs whose operation is based on multicopper oxidase and laccase demonstrated good performance and stability in acidic conditions; in particular, the glucose oxidase–laccase EFC was studied at a pH value of 4.5. Therefore, optimal pH conditions can be achieved by introducing a buffer solution into the EFC chamber [67].

Xiao et al. [63] investigated EFCs considered as disposable systems, because the components can potentially be biologically degraded. These properties demonstrate the potential of EFCs in next-generation green power sources for a circular economy. They reported a detailed list of EFCs with a maximum power density greater than 1 mW cm^−2^ [66].

Finally, to improve the performance of EFCs, the main problems to be solved are to enhance the output power and the service life. The output current of EFCs can be improved by adding conductive materials, connecting electrodes with enzyme active centers by covalent or non-covalent bonds, and using various natural or artificial enzymes and electronic mediators [64]. The service life, at present the major obstacle restricting the application of EFCs, can be improved by the biotechnological preparation of artificial enzymes, the synthesis of materials and electrodes with better biocompatibility by biomimetic technology, and by improved electrode preparation methods.

## 4. Ion Exchange Membranes for BioFCs

**Figure 6:**
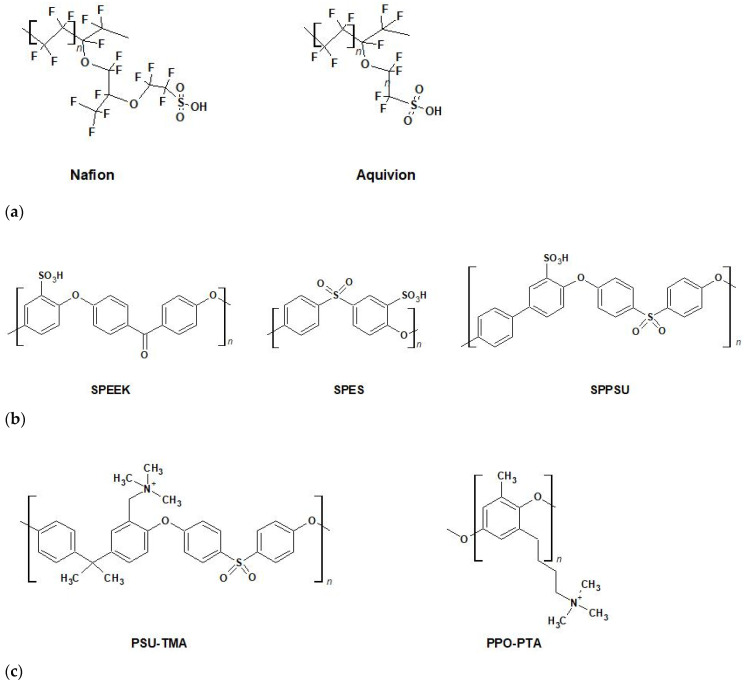
(**a**) Long side-chain perfluorosulfonic acid Nafion 1100 (*n* = 6.6) and short side-chain Aquivion 830 (*n* = 5.5). (**b**) Sulfonated aromatic polymers. (**c**) Anion exchange polymers: poly(sulfone trimethylammonium) (PSU-TMA) and poly(phenylene oxide pentyltrimethylammonium) (PPO-PTA).

BioFCs can also be subdivided according to the use or not of a membrane separator. Pure hydrogen cannot be used in membrane-less BioFCs given the risk of explosions without a separator between cathode and anode compartments. Furthermore, anaerobic conditions in the anodic compartment cannot be established, and the use of a single electrolyte without a separator increases the risk of parasitic electrochemical reactions. Current separators include foremost perfluorinated proton exchange membranes (PEMs), such as Nafion [102,103,104,105], Aquivion [106,107], and 3M [108]. Nafion and Aquivion exhibit a good endurance even under rigorous operating conditions [109,110]. The chemical structures of Nafion and Aquivion are shown in Figure 6a. The perfluorosulfonic polymers possess a poly(tetrafluoroethene) (PTFE) backbone and regularly spaced pendant perfluorovinyl ether side chains terminated by sulfonic acid groups. The side chain is longer in Nafion and shorter in Aquivion. The hydrophobic PTFE backbone provides excellent thermal and chemical stability, whereas the hydrophilic perfluorinated side chains act as proton sources.

The separator must also avoid the transfer of other redox-active species, such as sulfate, ammonia, ferricyanide, hydrogen peroxide, nitrate, and perchlorate, which degrade the Coulombic efficiency of the BioFCs, and some heavy metals that can alter the anodic microbes or enzymes and favor the proliferation of electrochemically inactive microorganisms [111]. The ion permeability and selectivity are thus important properties of separator membranes that can sensitively influence the power output of BioFCs: whereas cation-conducting separators such as Nafion and Aquivion block the passage of anions, anion-conducting separators impede the transfer of cations. 

Ramirez-Nava et al. [111] summarized the role and the most utilized membranes for BioFCs and in particular for MFCs. For dual chamber BioFCs, where the membrane introduces a challenge of cell configuration scaling up [68], expensive Nafion 117 is mostly utilized. The highly acidic sulfonic groups and fluorinated species can however be harmful for the biocatalysts and decrease their activity and lifetime [112]. In an attempt to solve the acidity problem, Schrenk et al. [113] mixed Nafion with quaternary ammonium bromide salts; the composite membrane exhibited similar mass transport properties compared to the pristine Nafion but showed a better selectivity to protons while becoming less selective against anions. In addition, Akers et al. [83] reported that modified Nafion helps to maintain the pH of the medium, thus becoming more compatible with biocatalysts. However, Nafion with tetrabutylammonium bromide salts remains expensive and non-biodegradable [114].

Recent studies about Nafion membranes in BioFCs demonstrate an important current density decay due to the interference of other cations, for example in phosphate buffers (e.g., K, Na, Mg) [115]. Along with protons, other high concentrated cations are transferred through the membrane. Nafion exhibits a selectivity and affinity to cations in the following order: Cs^+^ > Rb^+^ > Ba^2+^ > K^+^ > Mg^2+^ > Na^+^ > H^+^ > Li^+^ resulting in an increased conduction of most other cations rather than protons [116], leading to a reduction in conductivity and consequently an increased Ohmic drop and power density losses [117,118]. Furthermore, Gil et al. [39] demonstrated an increase in pH in the cathodic and decrease in pH in the anodic parts of the dual chamber cell separated by a Nafion membrane. They related this phenomenon to the slower rate of proton transfer through Nafion compared to the proton generation rate on the anode and proton expenditure rate on the cathode. In these conditions, the authors reported a constant anode potential, whereas the cathode potential decreased, resulting in an overall decreased power [118]. A pH stabilization is required by buffer solutions to guarantee the optimal enzymatic activity [117].

Anyway, perfluorinated ionomers present a relatively large permeability to gases (crossover) allowing oxygen to enter the anodic chamber, and they emit fluorinated products during use, which can denaturate enzymes and poison living organisms. Efforts have therefore been made to develop less expensive and toxic PEM with simpler synthetic processes, lower reactant permeability and without fluorine, especially sulfonated aromatic polymers (SAPs) [119,120,121,122]. SAPs (Figure 6b) include sulfonated poly(ether ether ketone) (SPEEK) [123,124], sulfonated poly(ethersulfone) (SPES) [125], sulfonated poly(phenyl sulfone) (SPPSU) [126], and sulfonated polysulfone (SPSU) [127]. However, SAPs suffer from poor hydrolytic stability under usual operative conditions, leading to a progressive deterioration of mechanical properties. Cross-linking treatments can improve their performance significantly [128,129].

Anion exchange membranes (AEMs) are now a fashionable alternative to PEMs, given the possibility to use non-noble metal electrocatalysts, including microbes and enzymes, as a result of the inherently faster kinetics of the ORR at higher pH [130,131]. Major interest in AEMFCs was generated by early reports of Varcoe and Slade [130,131,132,133] and commercial advances in materials and devices pursued by the Tokuyama Corporation [134]. There are now many groups investigating AEMFCs technology to break the constraints posed by acidic perfluorinated polymers and precious metal catalysts [134,135,136,137,138,139,140,141,142,143,144,145,146,147,148,149]. Various commercial aryl ether-based polymer backbones, such as poly(ether-ether ketone) (PEEK) [150,151] and poly(phenylene oxide) (PPO), were proposed [152,153,154] (Figure 6c), and some AEMs from the Holdcroft, Yan, and Bae groups were recently commercialized [155,156,157].

Many strategies were proposed to decrease the degradation of AEMs, which were related to the attack of positive ionic groups and labile bonds on the polymer backbone, such as ether links, by hydroxide ions [158] and to increase the long-term performance [142], including (i) change of the polymer backbone, especially without ether bonds [159,160,161], (ii) introduction of long side chains to separate the positive charge from the matrix [162,163,164], (iii) delocalization of the positive charge [149,165], (iv) cross-linking to improve the hydrolytic and mechanical stability [166,167,168], (v) formation of composite ionomers, generally obtained by mixing nanoparticles such as layered double hydroxides (LDH), SiO_2_, and TiO_2_ or forming nanoparticles via sol–gel routes [169,170,171,172,173,174]. Despite performances similar to PEMs, sometimes obtained with unrealistic testing conditions, the durability issues remain a primary challenge for the commercialization of AEMs [140], but some very encouraging results were reported recently [175].

Ex situ tests in important buffer solutions (phosphate, acetate, citrate) demonstrated that aromatic polymers functionalized with proton exchange or anion exchange moieties can be utilized assuring a higher conductivity than Nafion and good dimensional stability [79,112]. Amphoteric ionomers and bipolar membranes might be an interesting choice for use at intermediate pH values [176], but only very preliminary works in BioFCs were published [177].

## 5. Conclusions

In this short review, we summarized advancements of BioFCs without or with an ionomer separator. BioFCs, which can be further subdivided into microbial and enzymatic fuel cells, represent a long-term, affordable, accessible, and eco-friendly approach. Benefits of using BioFCs include the ability to derive sustainable energy from renewable sources. A second benefit is that they avoid the use of expensive catalysts and critical raw materials. Bacteria or enzymes are relatively inexpensive and easy to produce. For these reasons, their overall impact in terms of greenhouse gas emissions is low.

Although there have been major technological developments in this area, there is still a long way to go for their complete maturity. BioFCs are a nascent technology and compared to traditional FCs have lower electrochemical performances and power densities. Indeed, for the moment, they have only found application in powering remote monitoring devices that have low power needs. In the future, BioFCs could be a possible green solution from the perspective of sustainable development and the circular economy, but some challenging problems remain for their use on a global industrial scale with low cost.

Some advances in PEMs and AEMs development used as separators in BioFCs are presented at the end of the review. Membrane-less BioFCs suffer generally from a lower open circuit voltage because anaerobic conditions in the anodic compartment are difficult to provide, and the risk of parasitic electrochemical reactions is enhanced. Although perfluorinated membranes are mostly used, PEMs and AEMs based in aromatic polymers have recently been evaluated positively. The membrane durability is a however a tricky point that was recently improved quite a bit.

## Data Availability

Not applicable.
